# Semi-automatic standardized analysis method to objectively evaluate near-infrared fluorescent dyes in image-guided surgery

**DOI:** 10.1117/1.JBO.29.2.026001

**Published:** 2024-02-01

**Authors:** Tom H. Dijkhuis, Okker D. Bijlstra, Mats I. Warmerdam, Robin A. Faber, Daan G. J. Linders, Hidde A. Galema, Alexander Broersen, Jouke Dijkstra, Peter J. K. Kuppen, Alexander L. Vahrmeijer, Jan Sven David Mieog

**Affiliations:** a Leiden University Medical Center, Department of Surgery, Leiden, The Netherlands; b Amsterdam University Medical Center, Cancer Center Amsterdam, Department of Surgery, Amsterdam, The Netherlands; c Centre of Human Drug Research, Leiden, The Netherlands; d Erasmus MC Cancer Institute, Department of Surgical Oncology and Gastrointestinal Surgery, Rotterdam, The Netherlands; e Leiden University Medical Center, Department of Radiology, Leiden, The Netherlands

**Keywords:** fluorescence-guided surgery, image-guided surgery, standardization, image analysis, quantitative fluorescence imaging

## Abstract

**Significance:**

Near-infrared fluorescence imaging still lacks a standardized, objective method to evaluate fluorescent dye efficacy in oncological surgical applications. This results in difficulties in translation between preclinical to clinical studies with fluorescent dyes and in the reproduction of results between studies, which in turn hampers further clinical translation of novel fluorescent dyes.

**Aim:**

Our aim is to develop and evaluate a semi-automatic standardized method to objectively assess fluorescent signals in resected tissue.

**Approach:**

A standardized imaging procedure was designed and quantitative analysis methods were developed to evaluate non-targeted and tumor-targeted fluorescent dyes. The developed analysis methods included manual selection of region of interest (ROI) on white light images, automated fluorescence signal ROI selection, and automatic quantitative image analysis. The proposed analysis method was then compared with a conventional analysis method, where fluorescence signal ROIs were manually selected on fluorescence images. Dice similarity coefficients and intraclass correlation coefficients were calculated to determine the inter- and intraobserver variabilities of the ROI selections and the determined signal- and tumor-to-background ratios.

**Results:**

The proposed non-targeted fluorescent dyes analysis method showed statistically significantly improved variabilities after application on indocyanine green specimens. For specimens with the targeted dye SGM-101, the variability of the background ROI selection was statistically significantly improved by implementing the proposed method.

**Conclusion:**

Semi-automatic methods for standardized quantitative analysis of fluorescence images were successfully developed and showed promising results to further improve the reproducibility and standardization of clinical studies evaluating fluorescent dyes.

## 1 Introduction

Near-infrared (NIR) fluorescence imaging is an emerging imaging technique in the surgical field that uses light with a longer wavelength than visible light (the NIR-I spectrum; 700 to 1000 nm) to extract information from superficial and subsurface structures. As a result of the favorable optical properties of human tissue, photons with a wavelength in the NIR spectrum can travel further through human tissue compared with photons with wavelengths in the visible spectrum.^1^ NIR fluorescence imaging is used for perfusion imaging,^2^^–^^4^ the visualization of critical anatomic structures,^5^^–^^10^ and the identification of malignant tissue.^11^^–^^15^ In oncological surgery, fluorescent dyes are frequently conjugated to a tumor-targeting moiety, e.g., antibodies or peptides,^16^^,^^17^ to enable tumor-targeted imaging during surgery. Moreover, in fluorescence-guided liver surgery, the non-targeted fluorescent dye indocyanine green (ICG) is used to indirectly mark colorectal metastases.^18^^–^^20^ In contrast to tumor-targeted tracers, ICG accumulates in the tumor periphery, resulting in a typical fluorescent “rim-shaped” pattern.^18^^,^^21^ Although the two types of dyes accumulate in different regions, they are both valuable in identifying tumor tissue and assessing tumor margins.

NIR fluorescence-guided surgery has already shown great results in clinical research.^18^^,^^22^^–^^26^ However, further integration of fluorescence imaging in the surgical workflow is partly impeded due to the high variation in reproducibility of results between studies. To reduce this variation, standardization of the imaging protocols and quantification of the fluorescence signal are promising solutions that should be implemented. Standardization of the imaging protocols increases the comparability of the fluorescent signals between different images. By following such protocols even results of different fluorescence imaging systems can be compared.^27^^,^^28^ Quantification of the fluorescence signal enables the objective comparison of these signals. The implementation of standardized imaging protocols and quantifying fluorescence signals have gained interest in the field of perfusion imaging in the recent years.^29^^–^^32^ In contrast to perfusion imaging, standardized quantification methods or imaging protocols in oncological surgery have not yet been established. Most illustrative is the variation in imaging and drug administration protocols from one facility to another. The timing of ICG injection in colorectal liver metastases (CRLM) surgery varies between 1 to 14 days and the injected dose of ICG varies between 0.5  mg/kg and a total of 10 mg between institutions,^18^^,^^19^^,^^25^^,^^33^^–^^36^ resulting in outcomes of studies that are difficult to compare due to dissimilar conditions.

Koller et al.^37^ recently described the implementation of a standardized analytical framework to evaluate tumor-targeted fluorescent tracers. However, the framework lacks an objective method to analyze fluorescent signals as it is still based on manual subjective delineations of representative regions in fluorescence images, which is generally implemented in studies regarding fluorescence imaging in the oncological setting.^18^^,^^33^^,^^38^ The low reproducibility to select regions of interest (ROIs) in these methods results in higher inter- and intraobserver variabilities of fluorescence parameters, e.g., mean fluorescence intensity (MFI), tumor-to-background ratio (TBR), and signal-to-background ratio (SBR). This method could increase objectiveness by delineating in white light RGB images to eliminate the influence of the fluorescence intensities on the observer. Furthermore, the lack of a detailed description of the method used to select ROIs in these studies makes it difficult to replicate their results. Therefore, the aim of this study was to standardize the ROI selection and data analysis by developing and comprehensively describing a semi-automated analysis method for tumor-targeted and non-targeted dyes to compute the TBR and SBR to further objectify fluorescence measurements.

## 2 Methods

The proposed method is divided into three steps: (1) standardization of the imaging procedure, (2) standardized delineation of ROIs, and (3) automatic image analysis and data quantification. The steps of the proposed method are described in detail in this section.

### 2.1 Imaged Specimens

**Fig. 1:**
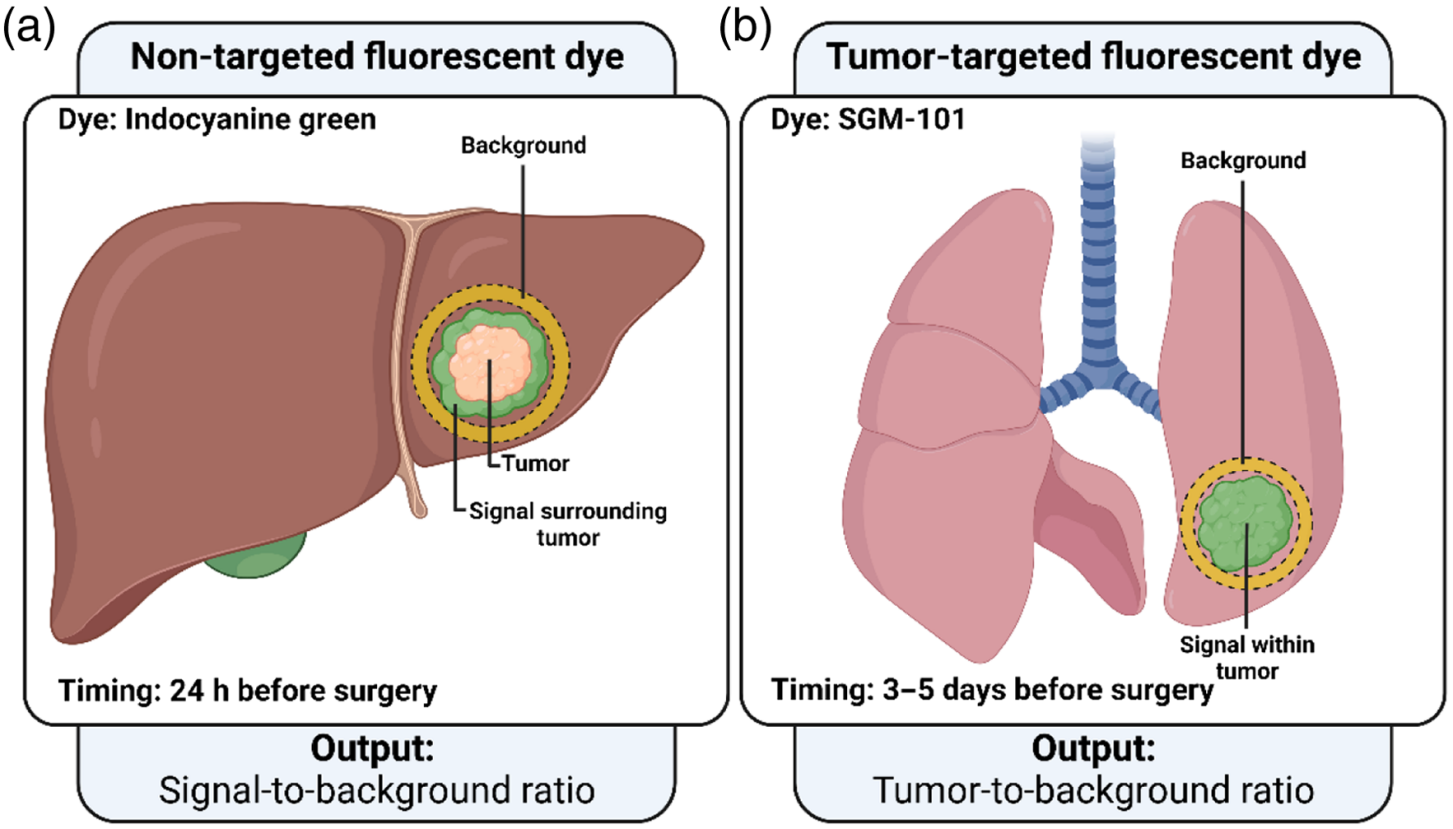
Difference between SBR and TBR. Fluorescence-guided surgery of CRLM can be performed with ICG, which accumulates around the tumor (a). Evaluation of the performance of ICG is performed by dividing the fluorescence intensity (signal) around the tumor by background fluorescence. Fluorescence-guided surgery of CLM can be performed with the tumor-targeted dye SGM-101, which accumulates inside the tumor (b). Here, the tumor area and the signal fluorescence are represented by the same region. Therefore, for CLM, the TBR is computed.

As aforementioned, fluorescence patterns are different for non-targeted and for tumor-targeted dyes. Therefore, separate analysis methods were designed for both type of dyes. To evaluate the proposed analysis methods, two patient cohorts were included in this research with different fluorescence patterns (Fig. 1). The images used in this research were obtained from earlier clinical trials performed within our organization. These studies were approved by the medical ethical committee “Leiden-Den Haag-Delft.” Written consent was obtained from all study participants. The first cohort included patients who underwent surgery for CRLM and were injected with 10 mg of ICG (2.5  mg/mL) 24 h prior to surgery. This cohort was used to evaluate the analysis method for non-targeted dyes. The second cohort included patients who had undergone surgery for colorectal lung metastases (CLM) and was used to evaluate the methods for tumor-targeted dyes. They received doses of 7.5, 10, or 12.5 mg of the tumor-targeted dye SGM-101, a carcinoembryonic antigen-targeted antibody specifically delineating colorectal malignancies. The drug was administered over 30 min, 3 to 5 days prior to surgery.

### 2.2 Imaging Procedure

To acquire images, the resected specimens were collected directly after surgery and transported to the Department of Pathology. Upon arrival, ink was applied to the resection plane by the pathologist to maintain the correct orientation of the resection plane with respect to the tumor. Directly after inking or after fixation of the specimen with formalin, the specimens were cut into ±5 mm thick tissue slices (i.e., bread loaves). The bread loaves were imaged on both sides in the PEARL Trilogy Small Animal Imaging System (LI-COR Biotechnology, Lincoln, Nebraska, United States). The PEARL imaging system standardizes factors affecting the measured fluorescence intensity (i.e., ambient light, the distance between the camera and imaged tissue, and the angle between the incoming light and the imaged surface) and is suitable for imaging fluorescent dyes around 700 and 800 nm.^38^^–^^40^ The parameters exposure and acquisition time are automatically set by the system, but it does not auto-gain the fluorescence intensities.^39^ A white light and a fluorescence image are both sequentially acquired while imaging with the system. The focus settings were adjusted so that the camera was focused on the specimen. The resolution was fixed at 85  μm, resulting in images of 1300×964  pixels.

### 2.3 Delineation of ROIs

After imaging was completed, the white light and fluorescence images were imported into QuPath, an open source software for image analysis.^40^ Brightness and contract settings of the white light image were set at a minimum of 0.00 and a maximum of 5.00 to standardize the delineation step. In contrast to general practice where ROIs are selected in the fluorescence image, the ROIs were manually selected in the white light image.^37^ By selecting ROIs in white light images, this step is objectified as the operators are not influenced by the fluorescence intensities. The two ROIs selected from the white light image were the macroscopic tumor and complete bread loaf. The tumor delineation was used to automatically select the ROIs, i.e., signal, tumor, and background fluorescence ROIs, in the fluorescence image. The selection of the bread loaf was used to solely include fluorescence data inside of the bread loaf of interest. Per lesion, the most central bread loaf was selected for ROI selection. To improve the accuracy of the manual delineations, the corresponding histopathological slides, the fluorescence image, and intraoperative images were used side-to-side with the white light image to provide additional spatial information. After completion, the delineations of the tumor and bread loaf were exported, and the fluorescence image was exported as an image with its original pixels. The images were exported to an OME TIFF file format to enable the import in the software where the quantitative image analysis was performed.

### 2.4 Image Analysis

The online available software for medical image analysis MeVisLab (version 3.4.1, MeVis Medical Solutions AG and Fraunhofer MEVIS—Institute for Medical Image Computing) was used to perform quantitative image analysis. Image processing pipelines can be designed in the framework of MeViSLab to evaluate images where all modules in the pipeline represent a step in the analysis process. Separate image processing pipelines were designed in MeVisLab for non-targeted and tumor-targeted fluorescence dyes.

#### 2.4.1 Pipeline I—SBR calculation for ICG around CRLM

**Fig. 2:**
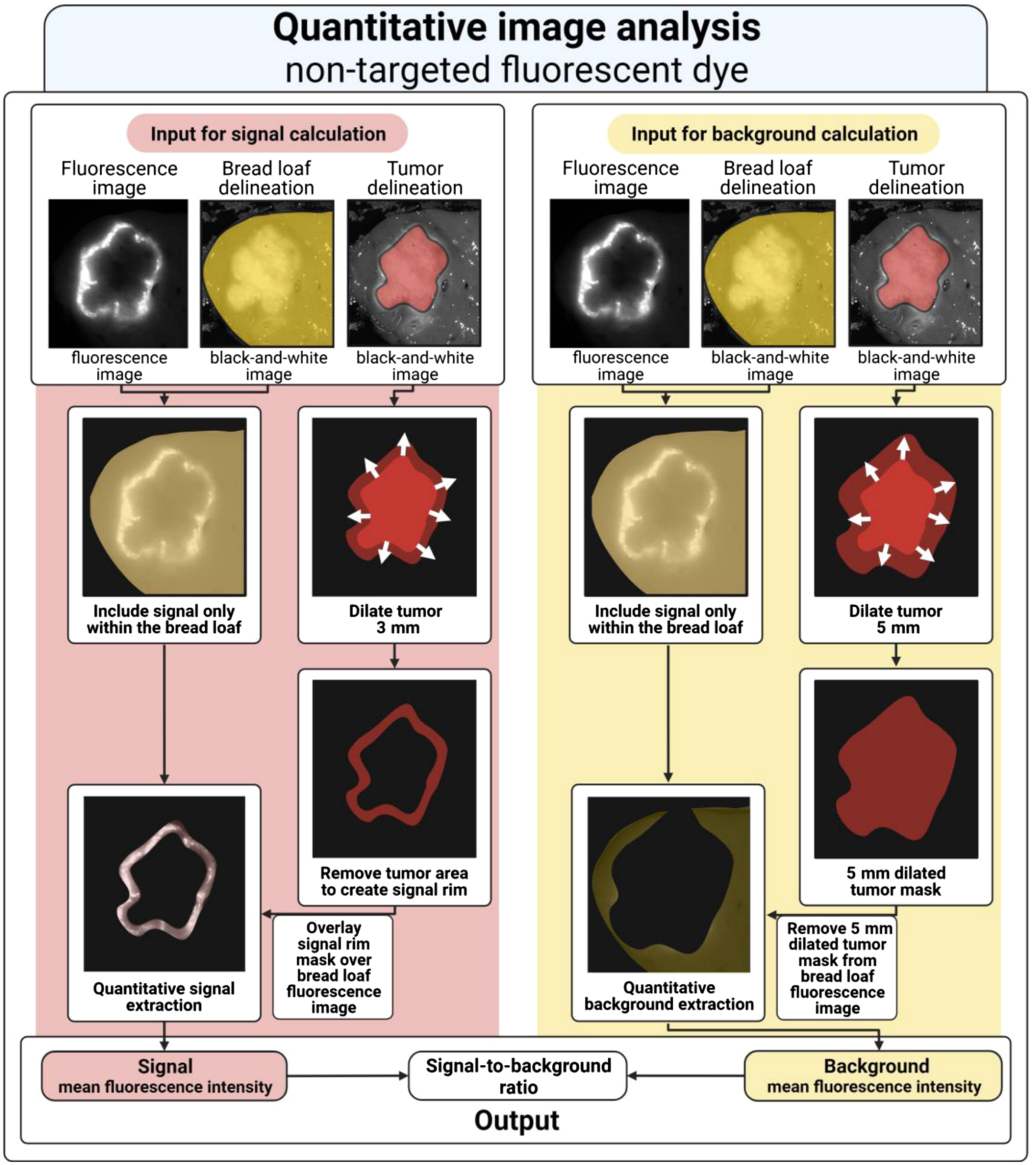
Overview of the quantitative image analysis model for non-targeted fluorescent dyes. The image analysis exists of two data pipelines: (1) signal calculation (red) and (2) background calculation (yellow). (1) The halo of the 3 mm dilated tumor is used as a mask on the bread loaf masked fluorescence image to calculate the MFI of the signal fluorescence. (2) The halo of the 5 mm dilated tumor is used as a negative mask on the bread loaf masked fluorescence image to calculate the MFI of the background fluorescence. Finally, the SBR is calculated by dividing the signal mean fluorescent intensity by the background mean fluorescent intensity.

First, both delineation images and the original-pixel fluorescence image were imported to MeVisLab. The delineated tumor and bread loaf images were processed to create masks (i.e., image filters that only allow the pixel values to pass, which lie within the mask) of the tumor and bread loaf. Thereafter, the signal fluorescence was computed by dilating the tumor mask by 3 mm. Subsequently, the tumor mask was subtracted from the enlarged mask and the MFI was computed for this region. The MFIs were computed with the use of automatically generated histograms with a bin size of 0.001 a.u. The background fluorescence was computed by dilated the tumor mask by 5 mm. This enlarged mask was subtracted from the bread loaf mask. The MFI of the resulting region was used as the background fluorescence. Here, the region up to 3 mm was chosen as signal fluorescence as this region provides relevant information about the delineation of the tumor. Healthy liver tissue from 5 mm from the tumor was chosen as background fluorescence to measure representative background fluorescence as ICG is spread heterogeneously in the liver. Moreover, this increased the chance that sufficient data were available to calculate the background fluorescence (e.g., at specimens with a small resection margin). The SBR was calculated by dividing the signal fluorescence MFI by the background fluorescence MFI (Fig. 1). An overview of this non-targeted dye image processing pipeline is illustrated in Fig. 2.

#### 2.4.2 Pipeline II—TBR calculation for tumor-targeted dyes

**Fig. 3:**
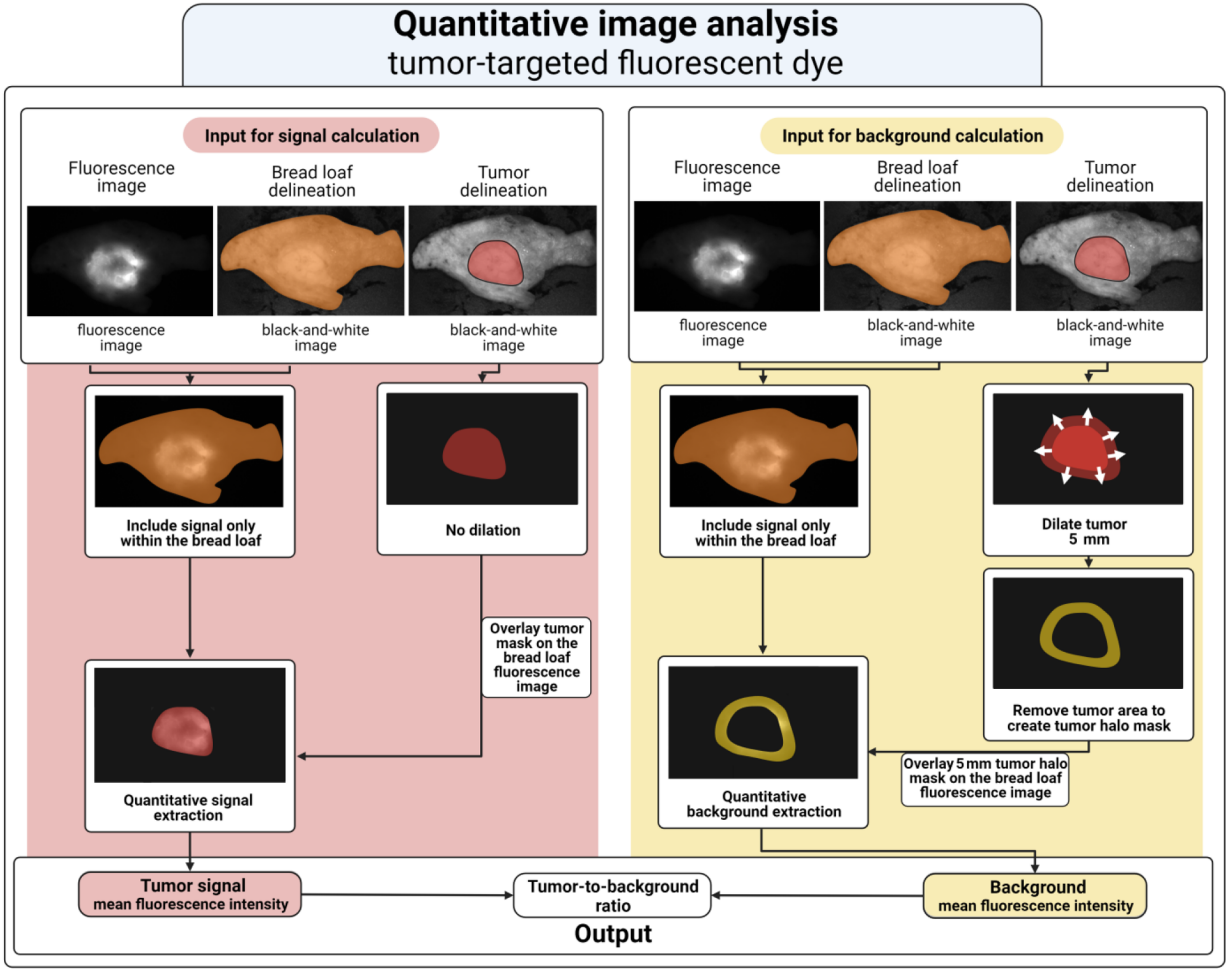
Overview of the quantitative image analysis model for tumor-targeted fluorescent dyes. The image analysis exists of two data pipelines: (1) signal calculation (red) and (2) background calculation (yellow). (1) The tumor delineation is used to calculate the MFI of the fluorescence only inside of the tumor. (2) The halo of the 5 mm dilated tumor is used as a mask on the bread loaf masked fluorescence image to calculate the MFI of the background fluorescence. Finally, the TBR is calculated by dividing the tumor signal mean fluorescent intensity by the background mean fluorescent intensity.

**Fig. 4:**
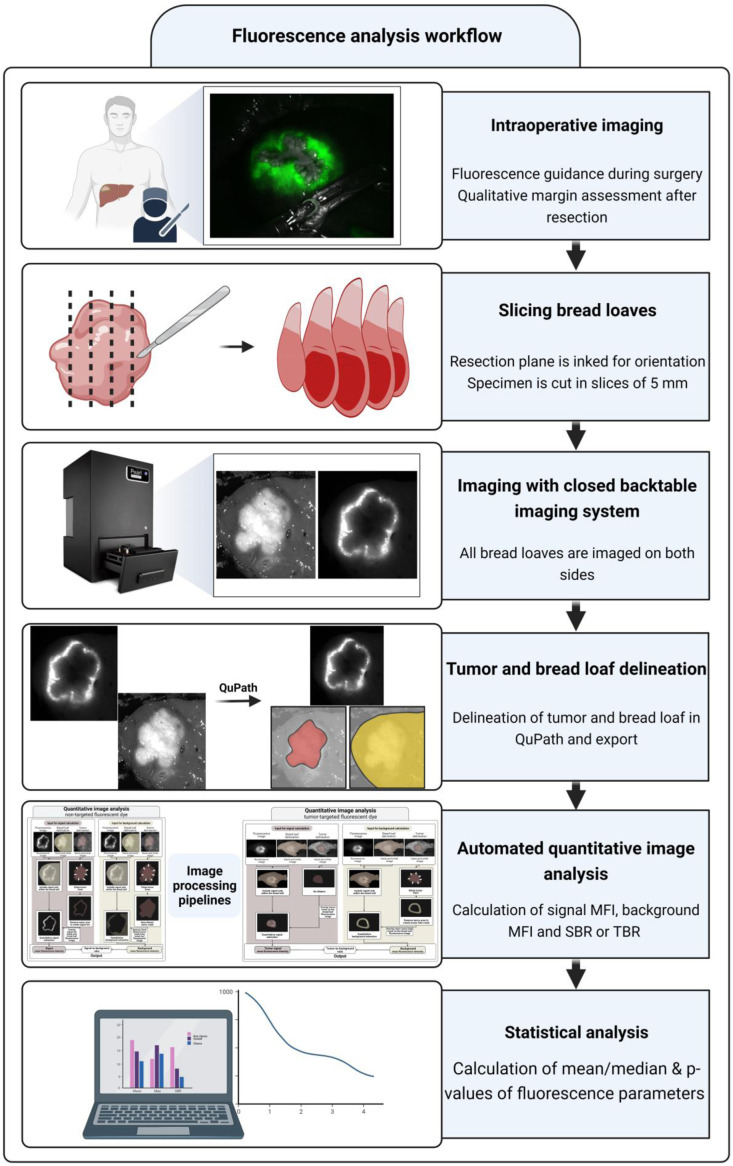
A schematic overview of the fluorescence analysis workflow. After intraoperative imaging, the resected specimen is imaged following a standardized protocol. The specimen is sliced in bread loaves and imaged with the PEARL imaging system. The PEARL imaging system sequentially acquires a white light and a fluorescence image of the specimen. In the white light image, the tumor and bread loaf are delineated. These delineations and the fluorescence image are used to collect quantitative data. The quantitative data can then be statistically analyzed in accordance with general practice.

For tumor-targeted dyes, the signal fluorescence was calculated by computing the MFI in the tumor with the use of the tumor mask. The background fluorescence was calculated by computing the MFI in the tissue within a reach of 5 mm surrounding the tumor. Thereto, the tumor mask was dilated 5 mm, and the original tumor mask was subtracted from the enlarged mask. The background fluorescence was then calculated by computing the MFI in this region. This region was chosen as background region for its clinical value as the fluorescence in the tumor is compared with the nearby background fluorescence to assess tumor-negative resection margins. The TBR was calculated by dividing the tumor MFI by the background MFI (Fig. 1). An overview of the tumor-targeted dye image processing pipeline is illustrated in Fig. 3. Furthermore, an overview of the complete proposed workflow is illustrated in Fig. 4.

### 2.5 Model Evaluation

To evaluate the proposed analysis method, it was compared with a conventional non-standardized manual analysis method. In the conventional method, the signal and background ROIs were selected manually in the fluorescence image based on the interpretation of the observer. Three experienced independent researchers (M.I.W., D.G.J.L., and R.A.F.) were asked to determine the SBR for the ICG cases and the TBR for the SGM-101 cases with the conventional method and the proposed standardized method. After 1 week, the observers repeated the measurements. The delineated ROIs and the resulting SBRs and TBRs were used to determine the inter- and intraobserver variabilities in the ROI selections and in the resulting TBRs and SBRs.

### 2.6 Statistics

Two different analysis strategies were used to evaluate the proposed analysis method. First, the inter- and intraobserver variations in the ROI selections were computed. Second, the inter- and intraobserver variabilities in the resulting TBRs and SBRs were calculated. The variation of the ROI selection was calculated to investigate the consistency of ROI selections. Hence, the results can be used to clarify potential differences or variation in the TBRs or SBRs. Variation in ROI selection does not necessarily have to result in variation in the resulting SBRs or TBRs. For statistical analysis, SPSS statistical package version 25 (SPSS, Inc., Chicago, Illinois, United States) was used. The Mann–Whitney U test was used to determine the statistical significance of differences in parameters between both methods.

#### 2.6.1 Inter- and intraobserver ROI variation

The variation in tumor and background ROIs was calculated by computing the dice similarity coefficient (DSC) of the ROIs.^41^ The DSC provides insight into the overlap of two different ROIs. The DSC ranges from 0 to 1, with a DSC of 1 indicating perfect overlap of two ROIs. For the interobserver ROI variation, the DSC was computed for three observer combinations (observer I versus II, observer II versus III, and observer I versus III). Per observer combination, a DSC was calculated for the four delineation session combinations (session I versus session I, session I versus session II, session II versus session I, and session II versus session II). The intraobserver variation in ROI selection was determined by computing the DSC between the delineations of the first and second delineation session per observer per bread loaf.

#### 2.6.2 Inter- and intraobserver TBR and SBR variability

The inter- and intraobserver variabilities were assessed with the intraclass correlation (ICC). To compute the ICC estimates and their 95% confidence intervals (CIs) for the interobserver variability, a two-way mixed-effects, absolute-agreement, mean-rating (k=3) model was used. For the intraobserver variability, a two-way mixed-effects, absolute-agreement, mean-rating (k=2) model was used. The interobserver ICC was calculated over both delineation sessions for both analysis methods. The intraobserver variability was computed once for all measurements for both analysis methods. The ICCs were rated poor (<0.5), moderate (0.5 to 0.75), good (0.75 to 0.9), or excellent (>0.9).^42^^,^^43^ The CIs were used to compare both methods and to make a statement about significant differences between both methods. When no overlap between the CIs was found, the result was interpreted as a statistically significant difference.

## 3 Results

In total, 10 bread loaves were available and included in each of the ICG and SGM-101 cohorts. For both cohorts, the signal MFI, background MFI, and corresponding TBRs or SBRs were calculated twice for all three observers, corresponding to the two delineation sessions. The results are presented in Table 1. The median [inter quartile range (IQR)] SBRs for the ICG cohort were 6.41 [4.85, 10.36] and 6.27 [4.58, 8.34] (p=0.749) for the proposed and conventional method, respectively. In this cohort, both the median [IQR] MFI of the signal and the background fluorescence intensity were significantly lower after calculation with the proposed method compared with the conventional method (signal MFI: (0.64 [0.35, 0.99] versus 1.14 [0.82, 1.59], p<0.001; background MFI: 0.10 [0.06 to 0.12] versus 0.16 [0.12 to 0.25], p<0.001). In contrast to the ICG cohort, the TBR after calculation with the proposed method was significantly lower compared with calculation with the conventional method in the SGM-101 cohort (3.16 [2.67 to 4.66] versus 4.20 [3.27 to 5.75], p=0.002). However, both the signal and the background MFIs were not statistically significantly different (signal MFI: 0.13 [0.10, 0.20] versus 0.13 [0.11, 0.21], p=0.384; background MFI: 0.05 [0.02, 0.06] versus 0.04 [0.02, 0.05], p=0.055).

**Table 1:** Median SBR and TBR and median mean fluorescence intensities.

	SBR			TBR			Mean signal/tumor fluorescence signal intensity (a.u.)			Mean background fluorescence intensity (a.u.)		
	Porposed method	Conventional method	*p*-value	Proposed method	Conventional method	*p*-value	Proposed method	Conventional method	*p*-value	Proposed method	Conventional method	*p*-value
ICG	6.41 [4.85, 10.36]	6.27 [4.58, 8.34]	0.749	NA	NA	NA	0.64 [0.35, 0.99]	1.14 [0.82, 1.59]	<0.001*	0.10 [0.06 to 0.12]	0.16 [0.12 to 0.25]	<0.001*
CRLM												
SGM-101	NA	NA	NA	3.16 [2.67 to 4.66]	4.20 [3.27 to 5.75]	0.002*	0.13 [0.10, 0.20]	0.13 [0.11, 0.21]	0.384	0.05 [0.02, 0.06]	0.04 [0.02, 0.05]	0.055
CLM												

Note: Depicts the median [IQR] SBR and TBR, mean signal/tumor fluorescence signal intensities (a.u.), and mean background fluorescence intensities (a.u.) of both patient cohorts for both evaluation methods. NA, not applicable.

* Statistically significant difference.

### 3.1 Inter- and Intraobserver Variation in ROI Selection

The inter- and intraobserver variation in ROI selection was described with the DSC. Table 2 shows the inter- and intraobserver variation in ROI selection in the ICG and SGM-101 cohorts for the proposed standardized automated analysis method and the conventional nonstandardized manual analysis method.

**Table 2:** Dice similarity coefficients.

	Intraobserver						Interobserver					
	Signal/tumor fluorescence ROI			Background fluorescence ROI			Signal/tumor fluorescence ROI			Background fluorescence ROI		
	Proposed method	Conventional method	*p*-value	Proposed method	Conventional method	*p*-value	Proposed method	Conventional method	*p*-value	Proposed method	Conventional method	*p*-value
ICG	0.85 [0.80, 0.89]	0.73 [0.55, 0.80]	<0.001*	0.95 [0.86, 0.97]	0.68 [0.34, 0.83]	<0.001*	0.75 [0.70, 0.82]	0.44 [0.27, 0.67]	<0.001*	0.90 [0.81, 0.94]	0.45 [0.28, 0.65]	<0.001*
CRLM												
SGM-101	0.95 [0.92, 0.96]	0.95 [0.94, 0.97]	0.496	0.87 [0.81, 0.90]	0.73 [0.63, 0.77]	<0.001*	0.92 [0.88, 0.94]	0.92 [0.89, 0.94]	0.591	0.76 [0.68, 0.84]	0.48 [0.37, 0.67]	<0.001*
CLM												

Note: Depicts the median [IQR] intra- and interobserver DSCs of the signal/tumor fluorescence ROIs and background fluorescence ROIs of both patient cohorts for both evaluation methods. A DSC of 1 corresponds to perfect overlap between both ROIs and 0 to no overlap.

* Statistically significant difference.

In the ICG cohort, the DSCs for both inter- and intraobserver variation analysis of the tumor as well as the background ROI selection were statistically significantly improved for the proposed method (all four: p<0.001). Furthermore, the inter- and intraobserver variations were statistically significantly higher for the proposed method in the SGM-101 cohort for the background ROI selection (both: p<0.001). The inter- and intraobserver DSCs for the tumor ROI of the SGM-101 cohort were similar for both methods (p=0.496 and p=0.591 for the inter- and intraobserver DSCs, respectively).

### 3.2 Inter- and Intraobserver Variability in SBR and TBR

The inter- and intraobserver variabilities in SBRs were computed for the proposed standardized automated analysis method and the conventional non-standardized manual analysis method for both patient cohorts. Table 3 shows the summary of the inter- and intraobserver variabilities in SBRs in the ICG and SGM-101 cohorts.

**Table 3:** Intraclass correlations.

	Intraobserver			Interobserver		
	Proposed method	Conventional method	Significant	Proposed method	Conventional method	Significant
ICG	0.98 [0.96 to 0.99]	0.80 [0.57 to 0.91]	Yes*	0.98 [0.95 to 0.99]	0.66 [0.24 to 0.86]	Yes*
CRLM						
SGM-101	0.98 [0.95 to 0.99]	0.98 [0.96 to 0.99]	No	0.93 [0.60 to 0.98]	0.96 [0.92 to 0.98]	No
CLM						

Note: Depicts the intra- and interobserver ICC coefficient [95% CI] of both patient cohorts for both evaluation methods. An ICC of 1 corresponds to perfect correlation and 0 to no correlation. When no overlap between the CIs was found the result was noted as a statistically significant difference.

* Statistically significant difference.

For the ICG cohort, the SBR calculations with the conventional method showed an interobserver variability (CI) with a poor to good ICC (0.66 [0.24 to 0.86]) over the two sessions. The proposed standardized method showed a statistically significantly higher ICC (CI) of 0.98 [0.95 to 0.99], scored as an excellent variability. The intraobserver variability of the conventional method (moderate to excellent reliable; 0.80 [0.57 to 0.91]) was significantly lower compared with the proposed standardized method (excellent reliable; 0.98 [0.96 to 0.99]).

For the SGM-101 cohort, the TBR calculations showed an excellent interobserver variability of the conventional method with an ICC of 0.96 [0.92 to 0.98]. The proposed standardized method showed a similar variability (moderate to excellent reliable; 0.93 [0.60 to 0.98]). The intraobserver variabilities for the conventional and proposed standardized method for the SGM-101 cohort were 0.98 [0.96 to 0.99] and 0.98 [0.95 to 0.99], respectively. Both labeled as excellent reliable.

**Fig. 5:**
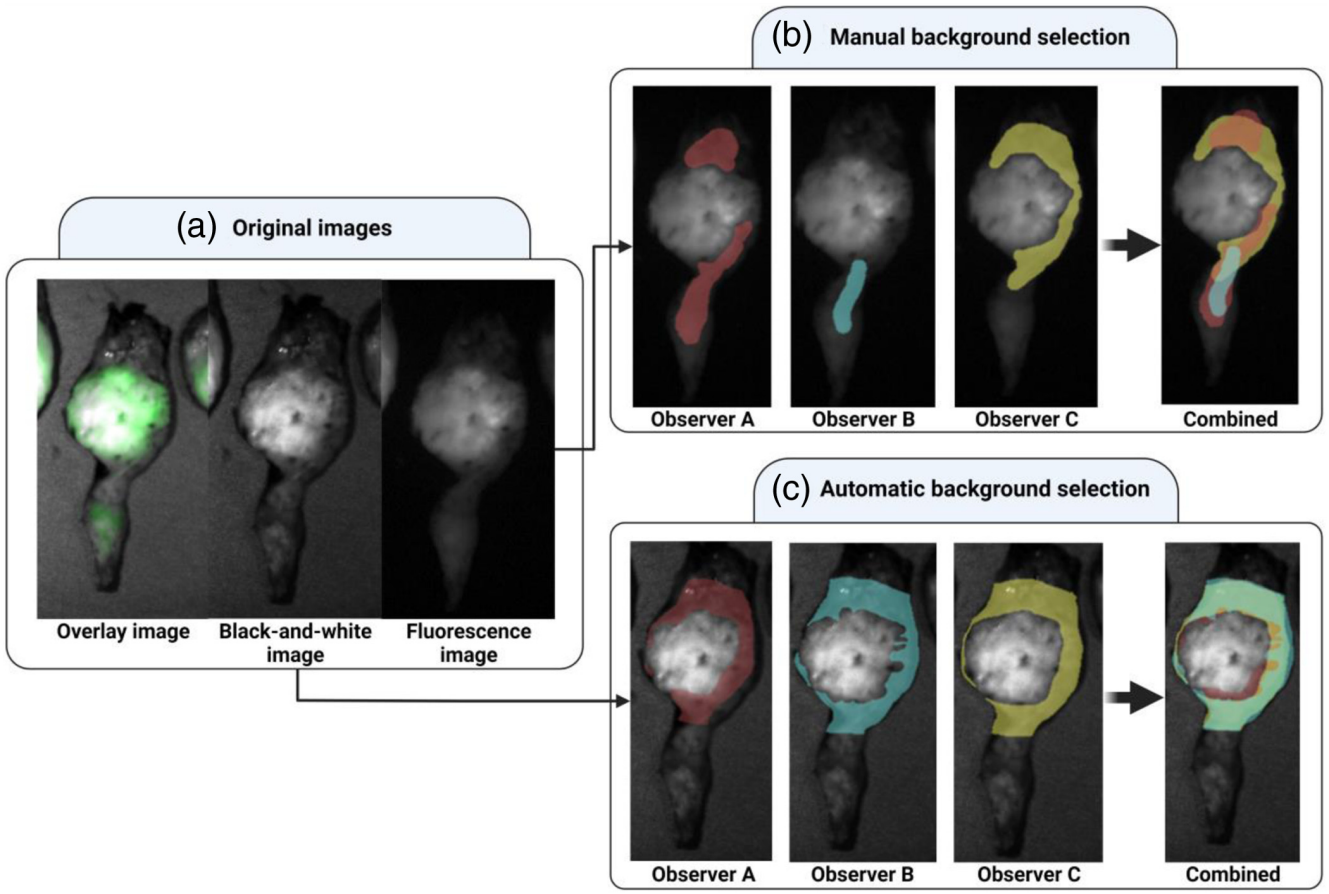
Background fluorescence selection by three different observers. (a) The overlay, black-and-white, and the fluorescence image. The patient received SGM-101 3 days prior to surgery. (b) The manual selections of the background region by three different observers and the combination of the three selections. The fluorescence image was used for these selections. (c) The automatic background region selections by the three observers and the combination of the three selections. The black-and-white image was used for these selections.

To illustrate the difference between the proposed standardized and conventional method to calculate TBRs, an example of the background selections of the three observers in a CLM SGM-101 case is shown in Fig. 5. The differences between these areas are large while in the test cohort the absolute agreement of the resulting TBRs remains high.

## 4 Discussion

We successfully designed a semi-automatic method incorporating in-house built software for standardized quantitative analysis of fluorescence images, which is, to the best of our knowledge, not described yet in literature. The method improved the inter- and intraobserver variabilities of ROI selection and SBR determination for the ICG cohort. This led to more objective and comparable fluorescence measurements within and between observers in this cohort. The SGM-101 cohort showed improved inter- and intraobserver variabilities in background ROI selection with the proposed method. This indicates that the standardized approach of our proposed method increases the objectivity of this manual operation. Therefore, our proposed analysis method could complement the analytical framework of Koller et al.^37^ by further improving its standardization. Wide implementation of this analysis method will potentially lead to increased efficiency in the adaption of new promising dyes, further accelerating the implementation of fluorescence-guided surgery in the field of oncological surgery.

This study was the first to unveil the effect and importance of standardization of the analysis method used to evaluate fluorescent dyes. By calculating the variation of the ROI selections, we were able to interpret the variation in the resulting SBRs or TBRs more in-depth, as completely different ROI selections do not necessarily have to lead to different TBRs or SBRs. However, implementation of the proposed analysis method led to a statistically significant decrease in MFIs and TBRs, suggesting the necessity of a slightly altered interpretation of parameters determined by our proposed analysis method.

The proposed analysis method includes reproducible and clearly definable steps that are generally applicable to studies evaluating the performance of fluorescent dyes in oncological applications. These are the imaging with the PEARL^®^ imaging system to completely standardize the imaging (i.e., ambient light, the distance between the camera and imaged tissue, and the angle between the incoming light and the imaged surface) and the automated selection of the ROIs with predefined dilation settings. The dilation settings can be modified for proposed applications of the method on samples of other tissue types or with other fluorescent dyes, e.g., the background fluorescence could be calculated over an area increased from 5 to 10 or 15 mm around the tumor for tumor-targeted dyes. The flexibility of these measurements increases the importance of transparent reporting of the exact settings used in this analysis method to be able to exactly repeat the calculations of the study. In this way, the transparency and reproducibility of studies that investigate the performance of new fluorescent dyes will be substantially improved.

There are still opportunities to improve our proposed analysis method, notwithstanding the improved variability and objectivity of the measurements. A limitation of our proposed method is the use of black-and-white images in which the delineations are performed. The absence of color in these images increases the difficulty to accurately delineate the tumor, potentially decreasing the inter- and intraobserver variabilities. Therefore, in black-and-white images of bread loaves in which no distinction can be made between malignant and healthy tissue, this proposed method is not applicable. Furthermore, the spread in the interobserver ICC for the tumor-targeted dye model was relatively large. This could be caused by minor differences in the manual tumor selection of the three observers. Delineations that do not include the complete tumor will increase the background fluorescence and therefore decrease the TBR. Overlaying Hematoxylin and eosin slides on the white light images to confirm the tumor boundaries could be a possible improvement to increase the accuracy of the delineations. Furthermore, the incorporation of artificial intelligence (AI) in the delineation step can further improve standardization of the analysis by minimizing human interpretations and interactions. Moreover, AI could be more time efficient as performing manual delineations is a time-consuming task.^44^^–^^47^ Therefore, future development should aim to automate the delineation step by implementing machine learning or deep learning into this step. QuPath has a feature to automate delineations, but this feature was not used in this research due to the limited dataset. To increase the availability of data to train AI models, our standardized imaging step must be widely implemented. This will accelerate the process toward automated delineations of tumor and bread loaves and fully automatic fluorescence image analysis. Ideally, the complete analysis method should be integrated into one software program, which is able to perform the delineation, data collection, and analysis steps. This will lead to fewer human interactions as images do not have to be exported and imported, and will simplify the analysis method for the user. Therefore, we are planning to start developing this software in our institute. Ultimately, this software could be integrated into the intraoperative workflow to support surgeons in decision-making, potentially decrease misinterpretation of fluorescent signals, and accelerate the learning curve of surgeons who start implementing fluorescence-guided surgery. Finally, we aim to enable real-time quantitative fluorescence-based intraoperative tumor margin assessment by implementing this surgery support software.

This study showed a decrease in TBR for the SGM-101 cohort when implementing the proposed method compared with the conventional method. This is an important outcome as this shows that when implementing our proposed method the resulting TBRs require a different interpretation compared with the TBRs determined with the conventional method. The ICG cohort showed statistically significantly improved variabilities for the ROI selections and resulting SBRs. These results suggest that eliminating human interpretation improves the variability as the manual selection of ROIs for non-targeted dyes is strongly subject to the interpretation of the fluorescence intensities. Therefore, based on the results of this study, we can conclude that the method to analyze the fluorescent signals is a step in the evaluation of fluorescent dyes that benefits from standardization and will improve research aiming to evaluate fluorescent dyes.

## 5 Conclusion

Our newly designed analysis workflow is a method that improves the objectivity and variability of fluorescence measurements for the analysis of fluorescent dyes for oncological surgical applications. Therefore, we advise to implement our proposed analysis method in future studies aiming to analyze the performance of fluorescent dyes. The increased objectivity of fluorescence measurements will lead to a decrease in discrepancies between studies and will pave the way for further implementation of fluorescence-guided surgery.

## Data Availability

Data and code developed in this study are available upon reasonable request to the corresponding author.
